# Telomere Length in Patients with Gestational Diabetes Mellitus and Normoglycemic Pregnant Women: a Systematic Review and Meta-analysis

**DOI:** 10.1007/s43032-023-01306-9

**Published:** 2023-07-25

**Authors:** Faustino R. Pérez-López, María T. López-Baena, Juan R. Ulloque-Badaracco, Vicente A. Benites-Zapata

**Affiliations:** 1 Universty of Zaragoza Faculty of Medicine, Domingo Miral s/n, 50009 Zaragoza, Spain; 2 Health Outcomes and Systematic Analyses Research Unit, Aragón Health Research Institute, San Juan Bosco 13, 50009 Zaragoza, Spain; 3 Escuela de Medicina, Universidad Peruana de Ciencias Aplicadas, Lima, Perú

**Keywords:** Birthweight, Gestational diabetes mellitus, Telomere length, Insulin, Glucose, Glycosylated hemoglobin

## Abstract

We performed a systematic review and meta-analysis of studies assessing telomere length in blood leukocytes or mononuclear cells in women with gestational diabetes mellitus (GDM) and normoglycemic pregnant women (NPW) and their infants. The review protocol was registered in PROSPERO (CRD42022300950). Searches were conducted in PubMed, Embase, LILACS, CNKI, and Wang Fang, from inception through November 2022. The primary outcomes were maternal and offspring telomere length. The Newcastle-Ottawa Scale was used to assess the quality of included studies. Random-effect meta-analyses were applied to estimate standardized mean differences (SMDs) and their 95% confidence interval (CI). The meta-analysis of four studies showed no significant maternal telomere length difference (SMD = −0.80, 95% CI: −1.66, 0.05) in women with GDM compared to NPW. In the sensibility analysis omitting one study with a small sample of women, the telomere length becomes significantly reduced in women with GDM (SMD = −1.10, 95% CI: −2.18, −0.02). GDM patients had increased glucose (SMD = 0.28, 95% CI: 0.09, 0.46) and glycosylated hemoglobin than NPW (SMD = 0.62, 95% CI: 0.23, 1.01) while total cholesterol, high-density lipoprotein cholesterol, low-density lipoprotein cholesterol, and triglycerides did not display differences between women with and without GDM. There was no significant difference in cord blood telomere length in offspring from women with GDM and NPW (SMD = 0.11, 95% CI: −0.52, 0.30). Cord blood insulin levels (SMD = 0.59, 95% CI: 0.33, 0.85) and birthweight (SMD = 0.59, 95% CI: 0.39, 0.79) were higher in offspring from pregnant women with GDM than in those from NPW. There were no significant differences in maternal and offspring telomere length between pregnancies with and without GDM.

**Supplementary Information:**

The online version contains supplementary material available at 10.1007/s43032-023-01306-9.

## Introduction

Gestational diabetes mellitus (GDM) is a glucose intolerance that appears during pregnancy and manifests by pancreatic insufficiency to neutralize the diabetogenic changes, increasing perinatal and maternal morbidity [1]. It is related to age, increased body mass index (BMI), basal estradiol, high-density-lipoprotein cholesterol (HDL cholesterol), and progesterone after ovarian stimulation [2–5]. In addition, family antecedents of insulin resistance or diabetes are associated with a higher risk of GDM. Magnesium deficiency is a risk factor for insulin resistance, secondarily diabetes mellitus type 2, and metabolic syndrome [6]. In women with GDM, there is evidence that magnesium participates in glucose metabolism inverse relationship between low magnesium intake and glucose metabolism [7]. A recent meta-analysis confirmed magnesium levels are lower in GDM cases than in control pregnant women [8]. Ambient air pollution exposure during the preconception period and the first half of pregnancy is associated with GDM risk [9]. Other factors involved in GDM genesis include a Westernized diet, genetic polymorphisms, advanced maternal age, polycystic ovary syndrome, and excessive weight gain during pregnancy [3, 10].

Increased GDM rate is associated with conceptions by assisted reproduction techniques (ARTs). In singleton pregnancies, the GDM risk is twofold higher in women receiving ART than in spontaneous conception [11]. During ovarian hyperstimulation cycles, lower estradiol levels are associated with GDM, and being highest with lower than 200 pg/mL [12]. Women receiving ARTs and blastocyst transfer have a higher risk of GDM than those conceiving spontaneously, and risk is significantly higher in women receiving day 5 blastocyst transfer than those receiving day 3 transfer [13]. During recent years, the deregulation of non-coding RNAs has also been related to metabolic disorders, including GDM and β-cell dysfunction [14].

Telomeres are terminal ends of chromosomes that are markers of cumulative cell damage in adults and during pregnancy [15, 16]. They are nucleoprotein complexes containing thousands of repetitive DNA sequences that protect the chromosomes from damage, senescence, and cell death [17, 18]. Genetic and environmental factors are involved in modulating telomere length. Also, several circumstances, including obesity, insulin resistance, and reduced physical activity, may contribute to shortening telomeres [19]. Short telomere length is associated with aging, diseases and metabolic disorders, social disadvantages, and unhealthy lifestyles [17, 20, 21]. However, the influence of telomeres on later life starts during pregnancy, and newborn telomere length influences future adult health [22–24].

In patients with type 2 diabetes mellitus, telomere length is inversely correlated with glucose level, including those with satisfactory glucose control [25]. A previous meta-analysis reported an association between diabetes mellitus and telomere length, influenced by region, age, diabetes type, BMI, and sex [26]. An umbrella review of observational studies reported that shorter telomere length might be associated with diabetes mellitus and other metabolic diseases [27]. However, the information concerning telomere length in pregnant women with GDM and their infants is limited or controversial. This systematic review and meta-analysis study the effect of GDM on maternal and offspring telomere length.

## Methods

### Protocol, Data Sources, and Search Strategy

This study followed the Preferred Reporting Items for Systematic Reviews and Meta-Analyses checklist for systematic review and meta-analysis [28]. The study protocol was registered in the International Prospective Register of Systematic Reviews (PROSPERO number: CRD42022300950). A comprehensive search syntax, using MeSH and free text terms, was developed for PubMed and appropriately adapted for other searched databases, including PubMed, Embase, LILACS (*Literatura Latino Americana e do Caribe em Ciências da Saúde*), CNKI (China National Knowledge Infrastructure), and Wang Fang. There were no restrictions during the search. The search MeSH terms included “telomere,” and “telomerase,” combined with “gestational diabetes mellitus,” “GDM,” or “diabetes pregnancy.” A description of the search terms and strategy is available in Table S1. We also hand-searched the reference lists of articles identified by this strategy, looking for additional papers. Studies published up to November 2022 in English, French, Portuguese, German, Spanish, or Chinese were considered for inclusion in this systematic review and meta-analysis without any restriction. We also hand-searched the reference lists of articles identified, looking for additional papers, and performed a search of the “grey literature” (e.g., medRxiv, and Grey Literature Report) to detect other potentially eligible investigations.

### Study Selection, Data Extraction, and Quality Assessment

**Fig. 1:**
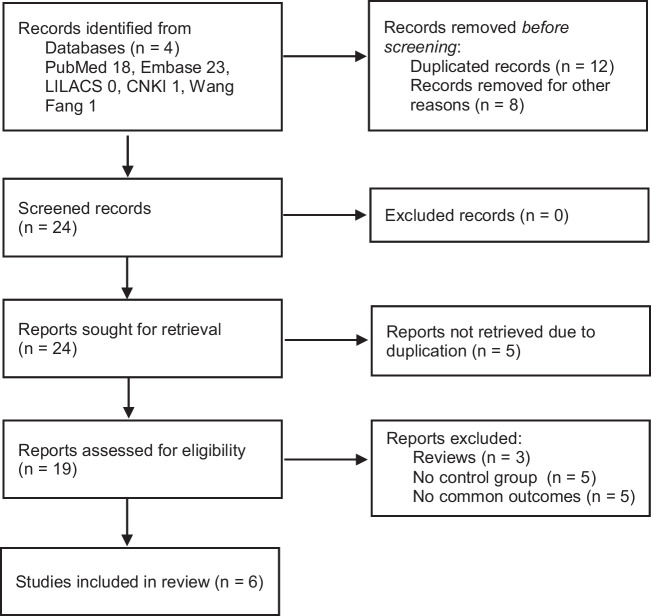
Flowchart of study selection

Two independent reviewers screened eligible studies based on their titles and abstracts. Studies were selected if they were original and peer-reviewed research reporting maternal or offspring telomere length in women with GDM and a control group of normoglycemic pregnant women (NPW) without pathology. After removing duplicates and scanning the titles and abstracts of articles, and those meeting the inclusion criteria were reviewed. Nineteen potentially relevant full texts were read to produce a final list of included studies. Any disagreements were resolved with discussion by adjudication to a third reviewer. Thirteen studies were excluded at this stage due to the lack of results comparing pregnant women with and without GDM (Fig. 1, Table S2). Six studies were included in the final analysis comparing maternal or offspring telomere length in pregnant women with and without GDM [29–34] (Table 1).

**Table 1:** Characteristics of six studies that evaluated maternal and/or offspring telomere length in women with GDM and normoglycemic pregnant women (NPW, control group): Location and period of study, GDM screening and diagnosis, sample size and age of women, BMI or weight gain and telomere length measurement methods

Authors	Location and period of study	GDM screening and diagnosis	Sample size and maternal age	BMI or weight gain	Telomere measurement and analytes
Cross JA, et al. [29]	Norwich and Ipswich, UK. February 2006 to 31 January 2008	Ipswich sample: WHO criteria. Norwich sample: ADA criteria. OGTT: 100-g.	GDM, *n* = 71 offsprings, age: 33.27 ± 5.28. Control, *n* =81 offsprings; age 31.52 ± 5.25.	Not reported	Cord blood telomere measured in mononuclear cells.
Gilfillan C et al. [30]	Victoria, Australia. Period of study: not reported.	GDM diagnosis: ADPS criteria. A 50-g glucose challenge at 26 weeks, and if positive a 75-g OGTT at 28 weeks.	GDM, *n* = 20. Control women, *n* = 18. Age: not reported.	Pregnancy BMI: GDM, 30.4 ± 10.4; control group, 28.2 ± 7.1, NS	Maternal and cord blood collected at delivery. gDNA measured by quantitative PCR
Harville EW et al. [31]	Seattle and Tacoma, Washington, USA. April 1998 to June 2002.	Screening at 24–28 weeks using a 50-g 1-h OGTT, according to the ADA, 2004. GDM criteria: 2 or more values higher in the 100-g OGTT.	GDM, *n* = 25; age: 30.9 ± 0.7. Control women, *n*= 50; age = 29.5 ± 0.5.	Pre-pregnancy BMI: GDM, 27.8 ± 7.5; control group, 22.4 ± 2.83.	Maternal leukocyte telomere length measured by quantitative PCR. Non-fasting glucose during intrapartum
Li P et al. [32]	Sichuan, Chengdu, China. October 2010 to April 2011.	GDM diagnosis: a 75-g OGTT at 24–28 weeks of pregnancy.	Sample size: GDM, *n* = 26; age: 31.8, IQR: 29.8, 37.4. Control group, 47 women; age: 30.2, IQR: 28.8, 32.2	Pre-pregnancy BMI: GDM, 21.6±3.2; control: 20.3±2.1. Weight gain: GDM: 14±4 kg; control group: 13±3 kg.	Maternal and cord blood leukocyte telomere length measured by quantitative PCR during intrapartum
Weng Q et al. [33]	Nanjing, China. October April 2014 and April 2015.	GDM diagnosis: A 75-g OGTT, according to the IADPSG criteria.	Sample size: GDM, *n* = 113; control group, *n*= 396, collected at 37.6±1.3 weeks (cases) and 37.8±1.9 weeks (control group).	Not reported.	Relative telomere length of genomic DNA from peripheral blood leukocytes. Fasting glucose in the last trimester of gestation
Xu J et al. [34]	Hangzhou, Zhejiang Province, China. Period of study: not reported.	GDM diagnosis: 75-g OGTT.	Sample size (infants): GDM, *n* = 82; control women, *n*=65.	Not reported.	Leukocyte telomere length in cord blood obtained at cesarean section.

*ADA* American Diabetes Association (ADA, 2004), *ADPS* Australian Diabetes in Pregnancy Society, *GDM* gestational diabetes mellitus, *gDNA* genomic DNA, *IADPSG* International Association of Diabetes and Pregnancy Study Groups, *IQR* interquartile range, *NS* not significant, *OGTT* oral glucose tolerance test, *PCR* polymerase chain reaction, *WHO* World Health Organization, *gDNA* genomic DNA

Included observational studies were eligible if they met the following criteria: population—pregnant women without pregestational and obstetric pathology not receiving any specific treatment; exposure—GDM diagnosis was according to an oral glucose tolerance test following international scientific organizations; comparator—NPW without GDM and any other obstetric or general pathology; outcomes—the primary outcome was telomere length measured in maternal or cord blood leukocytes or mononuclear cells. In all included studies, telomere length was measured by validated assays. Secondary outcomes were insulin, glucose- and lipid-related outcomes, and birthweight. Cord blood results were considered representative of infant values.

A data extraction form was used to obtain information on the following variables: country, sample size, age, clinical characteristics of pregnancies, methods used to assess the presence and absence of GDM, gestational age at telomere and/or telomerase measurement, and secondary measured outcomes. The following information was recorded from the selected literature: title, authors, year of publication, country, case-control basic information (number of subjects, age, and BMI), GDM screening procedure, maternal and offspring telomere length, insulin, and glucose-related and lipid-related outcomes.

For the meta-analyses, we collected mean and standard deviation measures. When the median and interquartile range (IQR) were provided, the mean was estimated by the formula *x* = (*a* + 2*m* + *b*)/4 using the values of the median (*m*), P25 and P75 (*a* and *b*, respectively), and the standard deviation (SD) was estimated using SD = IQR/1.35 [35]. When these were not provided or when mean and error measures were only presented in figures, we contacted the corresponding author to obtain specific information. Results reported as figures instead of numerical data were digitalized. Quality assessment was independently determined by two authors using the Newcastle-Ottawa Scale [36].

### Statistical Analyses

Because studies might have potential differences in recruitment procedures, phenotype baseline characteristics (including nutrition and physical activity), and laboratory measurements, we followed the DerSimonian and Laird random-effects model [37]. Continuous outcomes were planned as standardized mean differences (SMDs) to combine different measurement methods, with their corresponding 95% confidence intervals (CIs). The effect size is presented as SMD, and a *p*-value of < 0.05 was considered statistically significant. The Hedges’ *g* method was used to measure effect sizes, interpreting the magnitude of SMDs as small (0.20), moderate (0.50), or large (0.80) [38].

We evaluated statistical heterogeneity using the *χ*^2^, the *I*^2^ statistic, and the between-study variance using the *Tau*^2^. An *I*^2^ value of 0–30% defines low heterogeneity, 30–75% moderate heterogeneity, and >75% substantial heterogeneity [39]. A *p* < 0.1 for the *χ*^2^ defined the presence of heterogeneity, and a *Tau*^2^ > 1 defines the presence of substantial statistical heterogeneity. One-study leave-out sensitivity analysis was planned to test the robustness of the overall telomere length result [40]. A subgroup analysis compared telomere length in women living in China versus in the USA or Australia.

Statistical analyses were conducted using Review Manager (RevMan 5.3; Cochrane Collaboration, Oxford, UK).

## Results

### Eligible Studies

**Fig. 2:**
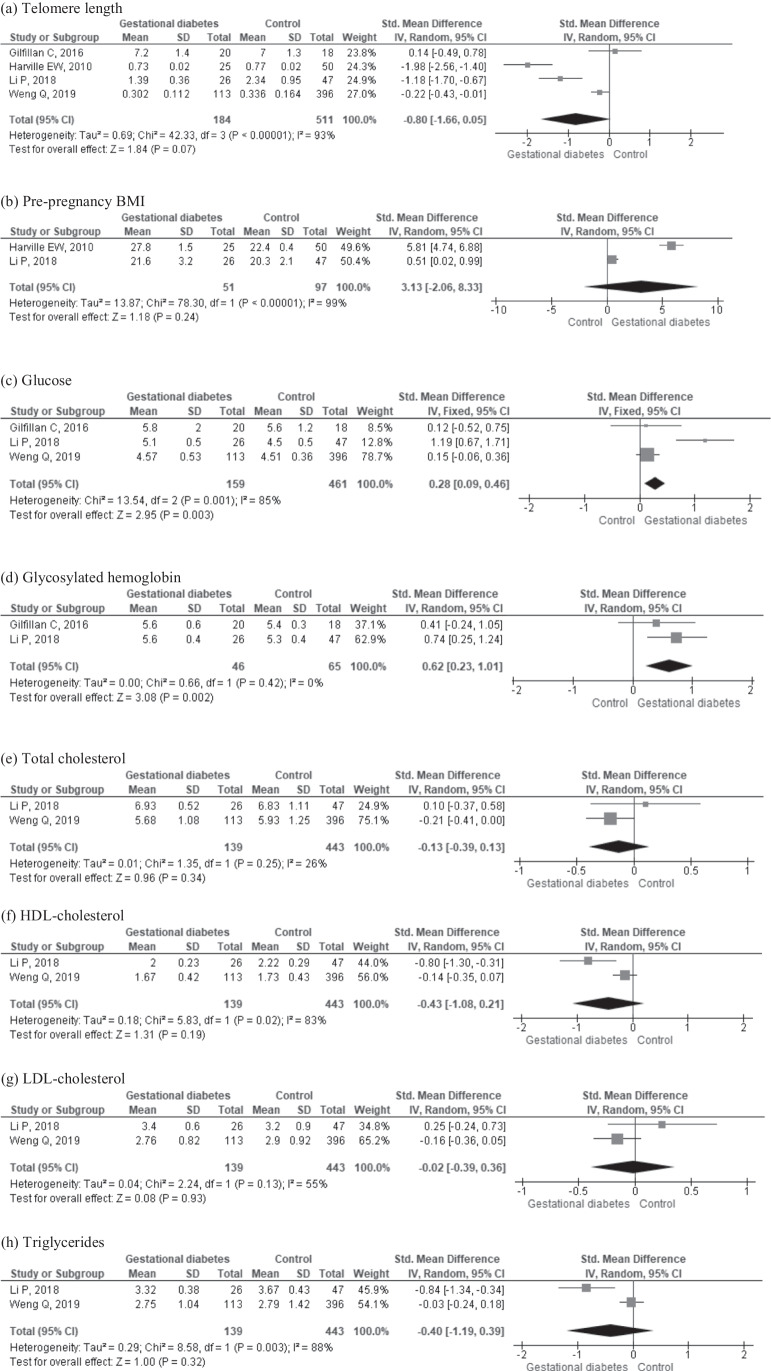
Forest plots of studies comparing maternal outcomes: **a** telomere length, **b** pre-pregnancy BMI, **c** glucose, **d** glycosylated hemoglobin, **e** total cholesterol, **f** HDL cholesterol, **g** LDL cholesterol, and **h** triglycerides in women with and without GDM

Table 1 displays information on the location and period of study, aims, GDM diagnosis criteria, number of participants, age, gestational age, and telomere measurement methods [29–34]. Pregnant women were screened for GDM at 24 to 32 weeks of pregnancy, using a 75-g oral glucose tolerance tests (OGTT) in five studies [29, 30, 32–34], and in one study a 100-g OGTT was performed with 100-g glucose [31] (Table 1). Telomere length was measured in maternal [30–33] and cord blood [29, 30, 32, 34]. In five studies, there was no information about clinical recommendations or treatments during the third trimester of pregnancy [29, 31–34]. In one study [30], 18 of 20 pregnant women with GDM were treated with insulin. Maternal BMI or weight results were reported in different ways: pre-pregnancy or pregnancy BMI, or weight gain during pregnancy (Table 1). Two studies provided information about pre-pregnancy BMI [31, 32] (Fig. 2c), one about pregnancy BMI [30], and another and weight gain during pregnancy [32]. Maternal blood sampling was performed intrapartum (non-fasting) [30, 32, 34] or during the third trimester of pregnancy [33, Table 1]. Only one study reported telomerase activity and telomere length [29].

### Meta-analyses, and Subgroup and Sensitivity Analyses

The meta-analysis of four studies (*n* = 695 participants) showed no significant difference in maternal telomere length in patients with GDM compared to NPW (SMD = −0.80, 95% CI: −1.66, 0.05; Fig. 2a, Table 1) [30–33]. Pre-pregnancy BMI [31, 32] did not display a difference in women with GDM and NPW (SMD= 3.13; 95% CI: −2.06, 8.33; 148 participants). Maternal glucose (SMD= 0.28, 95% CI: 0.09, 0.46; *n*= 620 participants, Fig. 2c) and glycosylated hemoglobin (SMD= 0.62, 95% CI: 0.23, 1.01; *n*= 111 participants, Fig. 2d) were higher in women with GDM compared to NPW, although sampling was not always under fasting conditions. There were no significant differences in maternal total cholesterol (Fig. 2e), HDL-cholesterol (Fig. 2f), LDL cholesterol (Fig. 2g), and triglycerides (Fig. 2h).

**Fig. 3:**
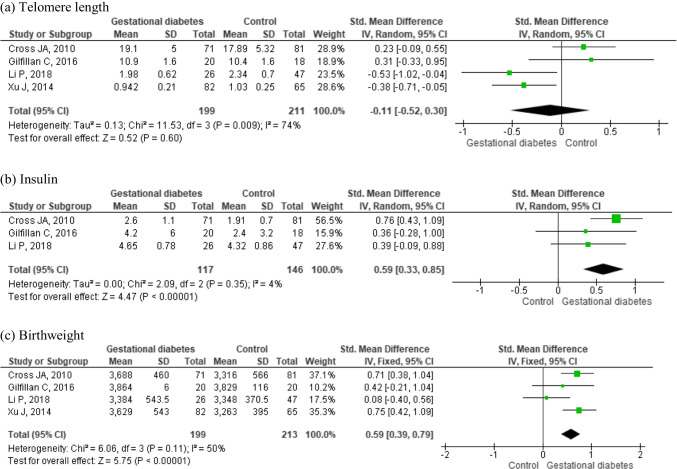
Forest plots of studies comparing offspring **a** telomere length, **b** insulin, and **c** birthweight from women with and without GDM

There was no difference between offspring telomere length from women with and without GDM (SMD = −0.11, 95% CI: −0.52, 0.30; *n* = 410 participants; Fig. 3a). Offspring insulin levels (SMD = 0.59, 95% CI: 0.33, 0.85; *n* = 263 participants; Fig. 3b) and birthweight (SMD= 0.59, 95% CI: 0.39, 079; *n* = 312 newborn; Fig. 3c) were significantly higher in infants from mothers with GDM compared to those from NPW. Other offspring outcomes were not available in at least two different studies.

The funnel plot analysis, with Begg’s correlation and Egger’s regression tests, was not calculated since there were not a minimum of ten studies. All maternal outcomes displayed a high heterogeneity (*I*^2^ > 75%), and offspring outcomes had moderate or high heterogeneity (Table S4). A one-study-leave-out sensitive analysis for maternal and offspring telomere length levels is presented in Table S4. Maternal telomere sensitivity analyses had high heterogeneity (*I*^2^ > 75%), and deleting the Gilfillan et al. [30] study with a small sample of women, telomere length was significantly lower in patients with GDM than in NPW (SMD= −1.10, 95% CI: −2.18, −0.02; Table S2A).

**Fig. 4:**
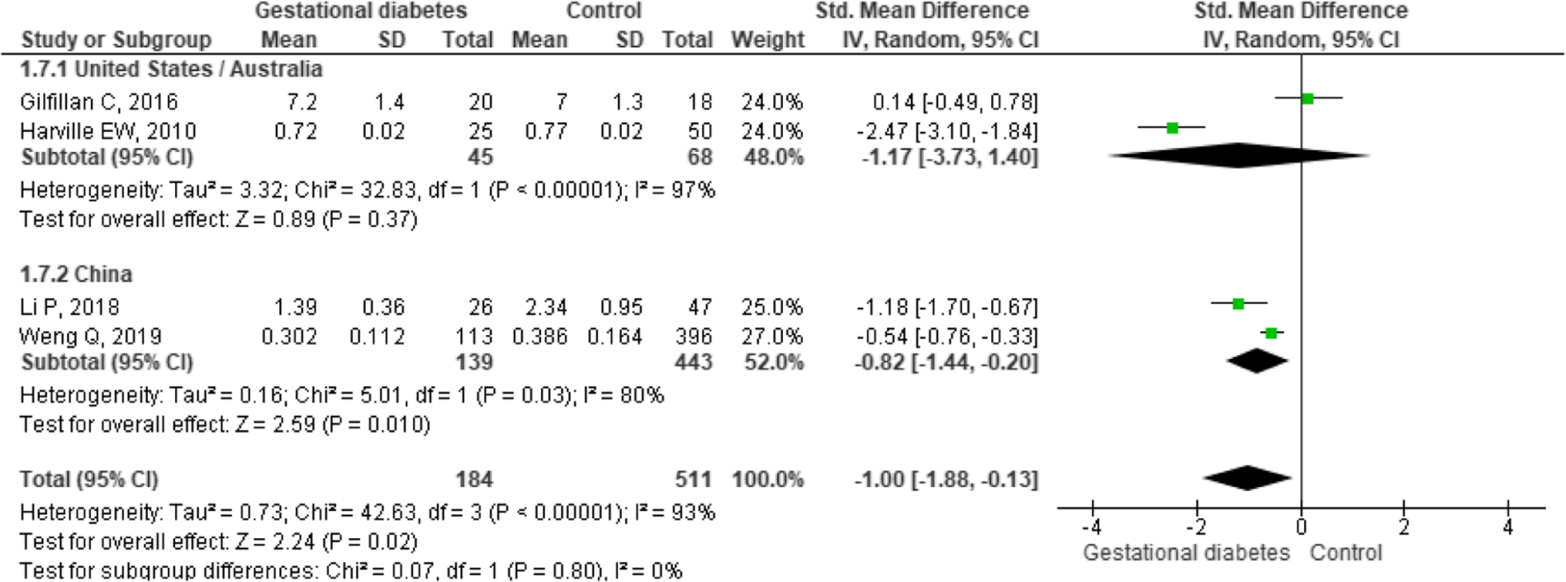
Sug-group analysis of maternal telomere length in women living in USA/Australia or China

The sub-analysis of women living in China compared to those living in the USA and Australia did not show a significant difference (*p* subgroup comparison 0.80, Fig. 4). Other subgroup options were not possible since the small number of studies.

## Discussion

This systematic review and meta-analysis report that maternal and offspring telomere length does not show difference between women with GDM and NPW. However, in the sensitivity analysis, maternal telomere length was shorter in GDM patients than in NPW after deleting one particular study with a small sample of women treated with insulin [36]. The offspring telomere sensitivity analysis did not show trend variations concerning the general meta-analysis. Insulin levels and birthweight were higher in the offspring of GDM patients than those from NPW. Maternal glucose and glycosylated hemoglobin levels were higher in patients with GDM than in NPW, and there was not difference in lipid metabolites, although some women were studied intrapartum.

### Maternal Telomere Length and GDM

Telomere length includes sequences of non-coding DNA at the eukaryotic chromosome ends that maintain genomic stability (Blackburn [17]). The length is inherited from maternal and paternal contributions [17, 41]. Telomere shortening is associated with oxidative stress, biological aging, metabolic disorders, diabetes, age, BMI, and world region of residence [25, 26]. Telomerase enzyme controls telomere integrity and includes a telomerase reverse transcriptase that adds the telomeric DNA repeats at the end of chromosomes, and a telomerase RNA component that serves as a template for telomeric DNA synthesis [42]. Telomerase is essential to maintain telomere length during human development. The high telomerase activity during pregnancy can neutralize intracellular inflammation, protecting telomeres in newborns [43, 44]. However, after birth its presence is limited to stem and germ cells, while somatic cells have no telomerase activity [13].

Trophoblast telomerase expression is lower in diabetic cases than in normal trophoblast; however, those differences are not detectable in cord blood leukocytes [45]. The trophoblastic equilibrium between telomere and telomerase may be altered in GDM patients, increasing the risk of metabolic disorders in the offspring of those pregnancies. The absence of difference in maternal telomeric length observed in our study might be explained by the action of telomerase during the embryonic period, as this enzyme restores telomeres and is more active during the embryonic formation phase [46]. However, telomerase activity has not been studied in women with GDM, probably due to the lack of precise measurement methods [47].

Telomere length reflects glucose metabolism alterations, and diabetic patients with better plasma glucose levels have longer telomere lengths [48, 49]. Insulin-dependent diabetes patients have shorter telomere lengths than non-diabetic subjects [50, 51]. Telomere length is also short in girls aged 9 to 16 years exposed to GDM in utero [52]. Some diet ingredients are involved in oxidative stress and inflammation processes that alter telomere length in different glucose metabolic alterations [48]. Although our meta-analysis of four studies with high heterogeneity demonstrated no significant difference in maternal telomere length using the random-effect model. However, in the sensitivity analysis deleting one study with a small sample of women treated with insulin and a moderate risk of bias [30], the other three studies displayed significantly lower SMD of maternal telomere length in women with GDM than in NPW. This situation is, in some way, an inverse *p*-hacking effect that leads to non-significant results since a small study did not have enough potency to detect outcome differences [53, 54]. Further studies with a low risk of bias and sufficient pregnant women are needed to clarify the issue since the *p*-hacking has no definitive solution yet [55].

There is a negative association between BMI or weight gain and telomere length among younger subjects [56], and a meta-analysis reported that obesity is associated with short telomere length in healthy adults [57]. Our results showed no difference in pre-pregnancy BMI in pregnant women with GDM and NPW. Future well-designed studies should provide detailed outcomes to determine the relationship between weight gain and fat mass during pregnancy on telomere length during pregnancy in patients with GDM.

Our sub-analysis of maternal telomere length according to the world regions compared available studies reporting women living in China or the USA and Australia. This approach summarizes factors like ethnicity, lifestyle, nutrition, and healthcare that may converge into a global concept to study the clinical issue of telomere length in women with and without GDM [58, 59]. The sub-analysis shows a similar telomere trend in the two subgroups, suggesting that general factors, lifestyle, and environment do not contribute to maternal telomere shortening. Therefore, GDM would be the main factor involved in the telomere length results of the studied pregnant women. Other sub-analyses were not possible since the few available studies concerning telomere length in pregnant women with and without GDM.

The maternal glucose and glycosylated hemoglobin meta-analyses showed higher levels in patients with GDM than in NPW. However, some meta-analyzed studies reported non-fasting glucose, glycosylated hemoglobin, and lipid results during delivery. Experimental studies suggest that adequate metabolic management of glucose metabolism might reduce the risk of telomere shortening [60]. Only two studies reported glycosylated hemoglobin, total cholesterol, HDL cholesterol, LDL cholesterol, and triglycerides that do not provide sufficient information compared to the available literature on their metabolism. Furthermore, lipid metabolism during GDM can follow different trajectories [61] that require more detailed investigations.

### Offspring Telomere Length and GDM

In patients with type 2 diabetes, hyperglycemia and related metabolic changes, and increased progression of insulin resistance, accelerate telomere shortening [62, 63]. Cross et al. [29] did not find cord blood telomere difference, although they reported an increase in cord blood telomerase activity in type 1 and GDM cases. We found no significant difference in the offspring telomere length of mothers with GDM and NPW, suggesting some fetal protection from the placenta. Further studies are needed to determine telomere and telomerase activity in women with and without GDM. Although maternal insulin does not cross the placenta, maternal hyperglycemia and other nutrient alterations can stimulate the fetal pancreas to increase insulin production and an increased risk of adiposity in the offspring of mothers with GDM [64]. In pregnant women with uncontrolled diabetes, the placenta telomerase expression is reduced in diabetic patients, increasing the risk of metabolic alterations during pregnancy and in the posterior years [45]. The damage is likely produced in the syncytiotrophoblast, which has a significant role in nutrient delivery to the embryo [65].

### Limitations and Strength

Our meta-analysis has several limitations, mostly due to the few available studies, the spectrum of meta-analyzed GDM patients, pregestational and gestational weight gain, maternal body composition, and physical activity during pregnancy deserve to be studied in GDM. Bhatt et al. [66] reported that obesity and subcutaneous fat accumulation might contribute to the shortage of telomeres. In healthy subjects, obesity accelerates telomere shortening [57]. Perhaps both pregnancy and obesity share some common effects of fat mass on telomeres in pregnant women with GDM. In addition, the association between BMI and telomere length may be partially due to the chronic subclinical inflammatory status and higher leptin [67]. Despite limitations, this study has the strength of studying the available evidence concerning maternal and offspring telomere length in pregnant women with and without GDM.

## Conclusion

Maternal telomere length was not significantly different in patients with GDM compared to NPG, although a small sample study might confer a *p*-hacking effect. There was no difference in offspring telomere length, while insulin levels and birthweight were higher in cases of GDM than in NPW. Further studies are needed to validate telomere length as a marker for glycemic progression and fetal growth during the third trimester of pregnancy based on better-designed investigations to detect the effect of GDM treatments during the third trimester of pregnancy and maternal body weight and inflammatory markers.

### Supplementary Information

ESM 1 (DOCX 32 kb)

## Data Availability

The datasets generated during and/or analyzed during the current study are available in the main text, tables, figures, and the supplementary information supporting this manuscript.
