# Supplementary material for: Telomere Length in Patients with Gestational Diabetes Mellitus and Normoglycemic Pregnant Women: a Systematic Review and Meta-analysis
Source: Reprod Sci. 2023 Jul 25;31(1):45–55. doi: 10.1007/s43032-023-01306-9 (PMC10784358; doi:10.1007/s43032-023-01306-9)
Supplement: Supplementary file 1 — (DOCX 32 kb) [file 43032_2023_1306_MOESM1_ESM.docx]

**APPENDIX A**

**Title:** Telomere length in patients with gestational diabetes mellitus and normoglycemic pregnant women: A systematic review and meta-analysis

**Journal:** Reproductive Sciences

This Appendix contains four Supplementary Tables.

**Table S1** (Online Resource 1) Search results (PubMed, Embase, LILACS, CNKI, and Wang Fang until November 2022). No limitation regarding study period nor language restriction.

**Table S2** (Online Resource 2) Deleted studies and reasons for exclusion.

**Table S3** (Online Resource 3) Assessment of bias risk of included studies with the Newcastle-Ottawa Scale in the systematic review and meta-analysis, evaluating (A) the telomere length in women with and without gestational diabetes mellitus or telomere length in offspring from women with and without GDM.

**Table S4** (Online Resource 4) Sensitivity analysis (by excluding one study at a time) reporting (A) SMD of maternal telomere length in women with and without GDM; and (B) offspring telomere length from women with and without GDM and 95% confidence interval (CI), Z score, and *I*^2^.

**Table S1** (Online Resource 1) Search strategies (PubMed, Embase, LILACS, CNKI, and Wang Fang until November 2022

| **Search Engines** | **Abstracts (n)** | **Comments** |
| --- | --- | --- |
| Pubmed | 18 | - |
| Embase | 14 | - |
| LILACS | 0 | - |
| CNKI | 1 | - |
| Wang Fang | 1 | - |
| After deleting duplicates | 11 | None. |

**Search strategy in EMBASE cambiar**

#1 'telomere length'/exp OR 'telomere length' OR 'telomerase activity'

#2 'gestational diabetes mellitus'/exp OR 'GDM'

#3 #1 AND #2

#4 #3 AND ('article'/it OR 'review'/it)

**Search strategy in PUBMED**

("gestational diabetes mellitus"[MeSH Terms] OR ("gestational"[Tw] AND "diabetes"[Tw] AND "mellitus"[Tw]) OR "gestational diabetes mellitus"[Tw]) AND ((("telomerase"[MeSH Terms] OR "telomerase"[Tw] OR "telomerases"[Tw]) AND ("activable"[Tw] OR "activate"[Tw] OR "activated"[Tw] OR "activates"[Tw] OR "activating"[Tw] OR "activation"[Tw] OR "activations"[Tw] OR "activator"[Tw] OR "activator s"[Tw] OR "activators"[Tw] OR "active"[Tw] OR "actived"[Tw] OR "actively"[Tw] OR "actives"[Tw] OR "activities"[Tw] OR "activity s"[Tw] OR "activitys"[Tw] OR "motor activity"[MeSH Terms] OR ("motor"[Tw] AND "activity"[Tw]) OR "motor activity"[Tw] OR "activity"[Tw])) OR (("telomere"[MeSH Terms] OR "telomere"[Tw] OR "telomeres"[Tw] OR "telomere s"[Tw] OR "telomeric"[Tw]) AND ("length"[Tw] OR "lengths"[Tw]))).

**Table S2** (Online Resource 2) Deleted studies and reasons for exclusion

| **Authors** | **Reason of exclusion** |
| --- | --- |
| Biron-Shental T et al. 2015 | Study of placental biopsies from patients with diabetes |
| De Zegher F et al. 2017 | Study of small-for-gestational-age, appropriate-for-gestational-age or large-for-gestational-age infants |
| Hahn MC et al. 2021 | Reported results can not be used in meta-analysis (Mean geometric values) |
| Holmes DK et al. 2009 | Control group fetuses submitted to feticides. |
| Lin Y et al. 2019 | Telomere length measured during the first trimester of pregnancy |

Biron-Shental T, Sukenik-Halevy R, Naboani H, Liberman M, Kats R, Amiel A. Telomeres are shorter in placentas from pregnancies with uncontrolled diabetes. Placenta. 2015;36(2):199-203. doi: 10.1016/j.placenta.2014.11.011.

de Zegher F, Díaz M, Lopez-Bermejo A, Ibáñez L. Recognition of a sequence: more growth before birth, longer telomeres at birth, more lean mass after birth. Pediatr Obes. 2017;12(4):274-279. doi: 10.1111/ijpo.12137.

Hahn MC, Werlang ICR, Rechenmacher C, et al. Telomere length in healthy newborns is not affected by adverse intrauterine environments. Genet Mol Biol. 2021;44(4):e20200411. doi: 10.1590/1678-4685-GMB-2020-0411.

Holmes DK, Bellantuono I, Walkinshaw SA, et al. Telomere length dynamics differ in foetal and early post-natal human leukocytes in a longitudinal study. Biogerontology. 2009;10(3):279-84. doi: 10.1007/s10522-008-9194-y.

Lin Y, Zhu Y, Wu J, Hinkle SN, et al. A prospective study of leukocyte telomere length and risk of gestational diabetes in a multiracial cohort. Epidemiology. 2019;30 Suppl 2(Suppl 2):S10-S16. doi: 10.1097/EDE.0000000000001081.

**Table S3** (Online Resource 3) Assessment of bias risk of included studies with the Newcastle-Ottawa Scale in the systematic review and meta-analysis, evaluating the telomere length in pregnant women with and without gestational diabetes mellitus or telomere length in offsprings from women with and without GDM

| **Study [reference]** | **Selection** | | | | **Comparability of exposed women and controls** | **Exposure** | | | **Total Score** |
| --- | --- | --- | --- | --- | --- | --- | --- | --- | --- |
|  | *Adequate definition of cases* | *Representativeness of cases* | *Selection of controls* | *Definition of controls* |  | *Ascertainment of exposure* | *Same method of ascertainment for exposed women and controls* | *Non-response rate* |  |
| Cross JA, 2010 [29] | * | 0 | * | * | * | * | * | * | 7 |
| Gilfillan C, 2016 [30] | 0 | * | * | * | * | * | * | 0 | 6 |
| Harville EW, 2010 [31] | * | * | * | * | * | * | * | * | 8 |
| Li P, 2018 [32] | * | * | * | * | * | * | 0 | * | 7 |
| Weng Q, 2019 [33] | * | * | * | * | * | * | * | * | 8 |
| Xu J, 2014 [34] | * | * | * | * | * | * | * | * | 8 |

**Supplementary Table S4** (Online Resource 4) Sensitivity analysis (by excluding one study at a time) reporting (A) SMD of maternal telomere length in women with and without GDM; and (B) offspring telomere length from women with and without GDM and 95% confidence interval (CI), Z score, *p*, and *I*^2^

1. Maternal telomere length sensitivity analysis

| **Studies [references]** | **SMD [95% CI]** | **Z** | ***p*** | ***I* ^2^ (%)** |
| --- | --- | --- | --- | --- |
| All studies included | -0.80 [- 1.66, 0.05] | 1.84 | 0.07 | 93 |
| Gilfillan C, 2016 [30] | -1.10 [- 2.18, -0.02] | 2.00 | 0.05 | 95 |
| Harville EW, 2010 [31] | -0.42 [-1.09, 0.24] | 1.24 | 0.21 | 85 |
| Li P, 2018 [32] | -0.68 [-1.79, 0.43] | 1.19 | 0.23 | 94 |
| Weng Q, 2018 [33] | -1.01 [-2.16, 0.13] | 1.74 | 0.08 | 93 |

1. Offspring telomere length sensitivity analysis

| **Studies [references]** | **SMD [95% CI]** | **Z** | ***p*** | ***I* ^2^ (%)** |
| --- | --- | --- | --- | --- |
| All studies included | -0.11 [- 0.52, 0.30] | 0.52 | 0.60 | 74 |
| Cross JA, 2010 [29] | 0.26 [- 0.67, 0.15] | 1.26 | 0.21 | 56 |
| Gilfillan C, 2016 [30] | -0.21 [-0.68, 0.27] | 0.86 | 0.39 | 80 |
| Li P, 2018 [32] | 0.02 [-0.45, 0.49] | 0.08 | 0.93 | 75 |
| Xu J, 2014 [34] | 0.00 [-0.52, 0.52] | 0,00 | 1.00 | 73 |
